# Strategic corporate entrepreneurship practices in financial services firms: the role of organizational factors

**DOI:** 10.1007/s43546-022-00306-2

**Published:** 2022-08-13

**Authors:** Belén Casales Morici

**Affiliations:** Department of Business Studies, Uppsala University, Uppsala, Sweden

**Keywords:** Strategic corporate entrepreneurship, Financial services firms, Organizational antecedents, Corporate Entrepreneurship Assessment Instrument, CEAI

## Abstract

The study explores a hitherto neglected area of strategic corporate entrepreneurship in services firms, the association of organizational factors with different strategic corporate entrepreneurship practices. It represents a first step toward understanding how internal organizational factors may spur different forms of strategic corporate entrepreneurship practices within services firms, especially those operating in the financial sector. Data for the study were collected through a survey study and the sample consisted of employees at a major insurance company in Sweden. The sample includes five offices placed in the north-eastern in Sweden and four offices ubicated in Stockholm. Using structural equation modelling, it was found that whereas organizational factors such as *management support* and *reward/reinforcement* have a positive effect on both incremental and discontinuous strategic corporate entrepreneurship practices, the organizational factor *work discretion* presents a differentiated effect. The study provides an important step toward understanding the internal factors that spur employees toward strategic corporate entrepreneurship practices in financial services firms. Moreover, the study develops a potential measure of incremental and discontinuous strategic corporate entrepreneurship practices. In terms of practical implications, the findings offer managers a solid foundation on which to base their decisions to create and improve an internal organizational environment that facilitates strategic corporate entrepreneurship practices.

**Supplementary Information:**

The online version contains supplementary material available at 10.1007/s43546-022-00306-2.

## Introduction

“Managers are supportive and help with guidance if there is a need to increase the pace, but otherwise, you have the freedom to perform your work as you want, it is ultimately up to your responsibility”...

“There was no structure for the development of entrepreneurial practices before, it was just a matter of staying afloat in the daily tasks. Now, intrapreneurship and innovation have become an important matter in the whole company”…

“Constantin”, an employee of a large and established financial service firm, gave me these accounts when I interviewed him. These comments not only highlight the efforts of financial services firms to increase the development of entrepreneurial practices but also the need to devise internal organizational mechanisms such as the support from managers and employee autonomy to stimulate those practices within the firm.

Several scholars have emphasized the importance of an organizational environment supportive of entrepreneurial practices within large and established firms (e.g., Antoncic and Hisrich 2001; Hornsby et al. 1993), including factors such as management support, work discretion, and reward/reinforcement (Antoncic and Hisrich 2001; Hornsby et al. 1993). Although since the 1990s, financial services firms have started significantly to improve its offerings digitalizing most business processes, the creation of an entrepreneurial organizational environment has not been easy to accomplish (Das et al. 2017). Especially since the financial crisis in 2008, they have been continuously challenged by new legislation aimed at market stability and competitiveness (e.g., Basel III[1], MIFID II[2], and PSD II[3]), which probably require bureaucratic and predominantly efficiency-oriented organizational mechanisms (Das et al. 2017). Consequently, the development and flow of new ideas within these firms have not been motivated among employees, causing that innovation in large and established financial services firms are often based on mere incremental improvements of their current offerings (Berry et al. 2006). However, in recent years they have been challenged by new players (e.g., large technology providers and financial start-ups) that continuously offer innovative services traditionally not provided by incumbent firms in the sector (Das et al. 2017). This increased pressure forced established financial services firms to channel efforts toward creating organizational mechanisms that could stimulate innovations.

While previous literature focuses strongly on obstacles to innovations in financial services firms, such as internal barriers (Das et al. 2017), consumer adoption barriers (Lee et al. 2003), cultural differences that result in barriers to implementing innovations (Singer et al. 2008) or the relationships between financial innovation and growth (Beck et al. 2016), less is known about what are the organizational drivers of strategic corporate entrepreneurship practices within large and established financial services firms. This study, therefore, takes a preliminary step toward addressing this important issue by asking what is the association of organizational factors such as management support, work discretion, and rewards/reinforcements with different strategic corporate entrepreneurship practices such as sustained regeneration, organizational rejuvenation, and strategic renewal.

The main objective of the study is to conduct a deeper examination of several internal organizational factors influencing incremental and discontinuous strategic corporate entrepreneurship practices to offer a more accessible way for established firms in building internal systems and processes that facilitate those practices. Specifically, the study explores the association between three organizational factors and three practices that strategic corporate entrepreneurship entails in their different natures (discontinuous and incremental).

The analysis of this association can offer firms important information and new insights to mitigate the inertia caused by regulations (Das et al. 2017). In this regard, the ability to simultaneously pursue both incremental and discontinuous strategic corporate entrepreneurship practices requires the design of contradictory internal organizational resources within the same firm (Tushman and O’Reilly 1996). Developing this ability allows the development of major transformations while facilitating the exploitation or the refinement of existing products or services.

Strategic corporate entrepreneurship practices involve strategy reformulation, reorganization, and purposeful redefinition of organizations to place firms on the path to competitive superiority or keep them in competitively advantageous positions (Guth and Ginsberg 1990). When firms exhibit strategic corporate entrepreneurship, innovations can happen anywhere and everywhere in the firm and may represent changes from companies’ past products, markets, organization structures, processes, capabilities, business models, or strategies (Hitt et al. 2011; Ketchen et al. 2007). The study contributes to both strategic corporate entrepreneurship and financial services firms’ literature in at least three ways. First, it furnishes novel empirical evidence of organizational antecedents supporting strategic corporate entrepreneurship practices in the often-overlooked financial service sector, thus moving away from the mono-sectorial manufacturing high-technology type of study commonly found in strategic entrepreneurship and innovation literatures. This allows scholars to develop more contextually sensitive theories and conceptual models (cf. Hughes and Mustafa 2017; Kyrgidou and Petridou 2011; Kantur 2016). Second, by exploring how the internal organizational environment can be designed to facilitate strategic corporate entrepreneurship practices, the study also provides important information for managers in financial services firms to create or improve internal organizational mechanisms that facilitate both incremental and discontinuous innovations. Third, as a methodological contribution, the study pioneers the clarification and differentiation of strategic corporate entrepreneurship practices, breaking them down into their different practices and nature, offering a foundation for developing a more generally applicable measure of the concept.

The article is structured as follows. First the related literature is described, and the conceptual framework and hypotheses are developed. Then, the study’s methodology is followed by the empirical analysis and the results. Finally, the study ends with a discussion, followed by the conclusions, the study limitations and suggestions for future research.

## Related literature

### Internal organizational factors and strategic corporate entrepreneurship practices in financial services firms

The societal and policy importance of innovation in financial services, call for empirical studies exploring the association between internal organizational factors and different innovation and renewal practices, i.e., the organizational drivers of these practices. A number of studies have shown and explored internal barriers that financial services firms face when they want to implement any organizational improvements and renewal within companies (e.g., Cooper and Edgett 2012; D’Este et al. 2012; Fattah et al. 2021; O’Reilly and Tushman 2013). Specifically, it has been found that barriers such as a restricted mindset, an inability to exploit new ideas, and a centralized organizational structure hamper further innovations (Das et al. 2017).

Several studies have mainly focused on innovation and different barriers. For example, consumer adoption barriers (Lee et al. 2003), organizational resistance toward firms' efficiency and innovation (Naveed et al. 2022), or cultural differences resulting in barriers to implementing innovations (Singer et al. 2008). Many relevant studies after 2008 focus on the impact of financial innovations on the market and customer behavior (e.g., Gerardi et al. 2010; Amin et al. 2008), the relationships between financial innovation and growth (Beck et al. 2016), or the effect of innovation such as the internet on a banks’ profitability (DeYoung et al. 2007). However, empirical research on internal organizational factors influencing innovation practices such as strategic corporate entrepreneurship practices in financial services firms is absent.

Although the importance of internal organizational factors supportive of those practices within companies has been empirically demonstrated in the literature (Antoncic and Hisrich 2001; Covin and Slevin 1991; Ireland et al. 2009; Kuratko et al. 2005; Zahra 1991), these studies have typically been placed in the context of new product and service, measured by broad measures such as the number of ideas implemented (Hornsby et al. 2009), external corporate venturing or strategic entrepreneurship without distinguishing among all the practices that it entails (Hughes and Mustafa 2017). Consequently, there is a need for further consideration of strategic corporate entrepreneurship as expressed in its different forms: sustained regeneration, organizational rejuvenation, and strategic renewal (Ireland et al. 2003; Luke and Verreynne 2006; Monsen and Boss 2009; Upson and Ketchen 2007). The extant literature in strategic corporate entrepreneurship is still in its infancy regarding differentiated measures and how these may relate to different aspects of the internal organizational environment. Some studies have used proxies such as entrepreneurial orientation and a scale based on Stevenson’s model of entrepreneurial management (Brown et al. 2001; Kyrgidou and Petridou 2011). However, these proxies are insufficient, as they do not capture the different forms that strategic entrepreneurship activities can take within the companies. Other studies (Monsen and Boss 2009) adopt the strategic entrepreneurship terminology but use the entrepreneurial orientation scale to measure the construct. Only one study developed a measure of strategic entrepreneurship construct based on content analysis of four semi-structured interviews and four focus groups studies (Kantur 2016).

## Conceptual framework

### The internal organizational environment

How employees perceive their working conditions determines the extent to which they experiment, demonstrate individual initiative, and use resources that have not been formally allocated to them (Morris et al. 2008). Therefore, entrepreneurial organizational factors have been identified in the literature as essential antecedents impacting strategic and entrepreneurial practices, especially in large and established firms (Antoncic and Hisrich 2001; Hornsby et al. 2009; Ireland et al. 2009). Hornsby et al. (2002) developed the Corporate Entrepreneurship Assessment Instrument (CEAI) scale, including five organizational factors: management support, work discretion, rewards/reinforcement, time availability, and organizational boundaries. Later, Hornsby et al. (2013) re-assessed the content, construct, and convergent validity of the CEAI. Only four factors remained after the analysis (without time availability), yielding an 18-item instrument to identify organizational antecedents based upon the original measure. A detailed description of these organizational factors is offered later when the hypotheses are developed.

### Strategic corporate entrepreneurship practices

There are two widely adopted notions of corporate entrepreneurship: (1) corporate venturing that focuses on the creation of new businesses within or outside the existing firm and (2) strategic corporate entrepreneurship. The last involves strategy reformulation, reorganization, and purposeful redefinition of organizations to place firms on the path to competitive superiority or keep them in competitively advantageous positions (Guth and Ginsberg 1990).

In recent efforts to produce an even more fine-grained understanding of strategic corporate entrepreneurship practices, the term innovation has also been added as a fundamental aspect of those practices (Kurakto and Audretsch 2009). In this respect, it is argued that when a firm exhibits strategic corporate entrepreneurship, innovations can happen anywhere and everywhere in the firm and may represent changes from companies’ past products, markets, organizational structures, processes, capabilities, business models, or strategies (Hitt et al. 2011; Ketchen et al. 2007).

According to the literature, the different forms in which strategic corporate entrepreneurship practices can be manifested within firms are sustained regeneration (new products and services offerings), organizational rejuvenation, strategic renewal, domain redefinition, and business model reconstruction (Morris et al. 2008).

- *Sustained regeneration* is, perhaps, the most widely accepted and recognized evidence of firm-level entrepreneurial activity. Firms that engage in sustained regeneration regularly introduce new products and services or enter new markets. The aim is to capitalize on latent or under-exploited market opportunities (cf. Covin and Miles 1999; Kurakto and Audretsch 2009; Morris et al. 2008).
- *Organizational rejuvenation* is the process, whereby the organization tries to sustain or improve its competitive standing by altering its internal processes, structures, and capabilities, aimed at enhancing the implementation of the firm’s strategy (cf. Covin and Miles 1999; Kurakto and Audretsch 2009; Morris et al. 2008). Morris et al. (2008) argues that organizational rejuvenation, in terms of changing existing activities in the value chain, mainly supports activities such as process and administrative innovations rather than product innovation. Similarly, Dess et al. (2003) show that firms also display entrepreneurship by changing processes and structures. In the financial sector, for instance, new activities such as the recent use of mobile apps for customers’ claims and the utilization of artificial intelligence (e.g., to answer customer queries or to better understand and compare insurance policy language), undoubtedly require organizational innovations in the form of new tasks, new administrative processes, structures, and capabilities.
- *Strategic renewal*^1^ refers to “the transformation of organizations through the renewal of the key ideas on which they are based” (Guth and Ginsberg 1990: 5). According to Covin and Miles (1999) and Morris et al. (2008), it describes new business strategies that significantly differ from past practices, i.e., radically or incrementally change the basis on which firms compete. In that sense, efforts toward strategic renewal focus on revising or replacing the company’s vision and strategy.
- *Domain redefinition* occurs when a company “proactively creates a new product–market arena that others have not recognized or actively sought to exploit” (Covin and Miles 1999, p. 54). Authors have also referred to domain redefinition as bypass strategy (Fahey 1989), market pioneering (Golder and Tellis 1993), whitespace marketing (Maletz and Nohria 2001), and the blue ocean strategy (Kim and Mauborgne 2005). Unlike the other forms of strategic corporate entrepreneurship, domain redefinition necessarily results in creating new businesses (Kuratko and Audretsch 2009).
- Finally, *business model reconstruction* refers to the entrepreneurial phenomenon in which a company designs or redesigns its core business model to improve operational efficiencies and differentiate itself from competitors (Kurakto and Audretsch 2009; Morris et al. 2008).

The study reported here focuses on three forms of strategic corporate entrepreneurship: sustained regeneration (e.g., new product and service offerings), organizational rejuvenation, and strategic renewal. The other two forms—domain redefinition and business model reconstruction—were not examined. First, companies in the financial services sector rarely create product-market positions unfamiliar to their competitors (Berry et al. 2006), which suggests that domain redefinition seldom occurs in the sector. Second, continuously challenged by legislation and regulations aimed at market stability (Das et al. 2017), insurance firms rarely engage in reconstructing their business model. Therefore, the frequency of these two forms of strategic corporate entrepreneurship is believed to be low, especially among large and established financial firms such as the analyzed in this study (Covin and Miles 1999; Morris et al. 2008).

To develop the items included in the dependent variables, it is assumed that strategic corporate entrepreneurship is a distinct and empirically verifiable set of organizational phenomena (Covin and Miles 1999). Consequently, practices aimed to rejuvenate or redefine organizations in terms of new products or services, organizational processes, and strategic orientation can be conducted in a discontinuous manner, energizing organizations radically, or incrementally through a series of regular minor adjustments.

### Discontinuous and incremental strategic corporate entrepreneurship practices

Discontinuous strategic corporate entrepreneurship practices involve significant alterations within the company along multiple dimensions, specifically concerning their products and services, organizational processes, structures, and strategic orientation. A company may conduct discontinuous strategic corporate entrepreneurship practices due to major changes in the environment, such as technology and customer demand, or because its primary market has matured or declined (Agarwal and Helfat 2009). It results from the search for new products and services and the discovery of new approaches to technologies that will probably result in new organizational routines and processes.

Because major transformations can pose significant difficulties due to the extent of change required, companies may also seek to renew themselves in incremental ways. Incremental strategic corporate entrepreneurship practices involve a series of regular minor adjustments in the firm, i.e., a gradual process that maintains the firm’s operations (Agarwal and Helfat 2009). When companies introduce new products or services incrementally, they are often connected to existing product and service portfolios. In this regard, the purpose of incremental strategic corporate entrepreneurship practices is to improve the existing skills and processes, applying extant knowledge in current domains.

Next, the organizational antecedents of strategic corporate entrepreneurship practices are explained; in what follows, hypotheses are interweaved. The analysis is based on specific theoretical pillars in the corporate and strategic entrepreneurship literature.

## Hypotheses development

### Management support

Burgelman’s (1983) seminal work prompted that the success of bottom-up entrepreneurial practices depends on managers’ receptiveness. In line with this, the support of managers has been recognized as the most critical organizational antecedent (Antoncic and Hisrich 2001; Hornsby et al. 2002; Kuratko et al. 1990; Moriano et al. 2011). It includes employees' perception about the commitment and support of their managers, the trust placed in them when innovative ideas are provided (Rutherford and Holt 2007; Schindehutte et al. 2000), as well as the perceived style of managers’ decision-making. In other words, it refers to the employees’ perceived trustworthiness to their companies in terms of detecting opportunities and willingness to develop novel ideas (Stevenson and Jarillo 1990). Therefore, it has been argued that managers should promote explorative behaviors among employees by encouraging them to solve problems in innovative ways and to seek opportunities proactively while enabling the firm to seek advantage in the marketplace (Villiers-Scheepers 2012).

Management support, therefore, plays a key role in encouraging employees to believe that innovation is expected from all members of the organization. The support from managers is manifested in a range of activities, including championing innovative ideas, recognizing people who articulate those ideas, providing the necessary resources or expertise (e.g., seed money to initiate projects), and institutionalizing entrepreneurial practices within the firm’s system and processes (Hornsby et al. 2002).

Several studies have supported these statements. For instance, Elenkov and Manev (2005) identify management support as one of the most important organizational factors in fostering entrepreneurial behaviors among employees. Fini et al. (2012) suggest that managers must create conditions for individuals to perceive entrepreneurial-related actions as desirable and feasible. Zampetakis et al. (2009) highlight that perceived organizational support positively affects individual entrepreneurial behavior. The above statements then lead to formulating the first hypothesis:

H1: Management support is positively related to incremental and discontinuous strategic corporate entrepreneurship practices.

### Work discretion

Work discretion is concerned with the degree of autonomy that employees have to decide how to perform their work most efficiently (Hornsby et al. 2002; Kuratko et al. 1990; Slevin and Covin 1990). Employees encouraged to decide how to achieve their goals will find more creative ways of doing so, being more willing to experiment and innovate (Hornsby et al. 2002; Sathe 1985; Stopford and Baden-Fuller 1994). At the same time, it opens up for the firm to examine a larger number of potentially attractive market-related opportunities (Ireland and Webb 2007). The autonomous and change-oriented behavior of employees in an organizational context is believed to promote innovative practices. It often involves the acts of speaking out, proactiveness, and challenging the status quo (Calisto and Sarkar 2017).

Furthermore, an organizational environment that is perceived with certain levels of autonomy can retain and exploit the innovative talent of employees, since a strong entrepreneurial motivation is a result of providing employees with a certain degree of freedom (Aube et al. 2007). Autonomy decentralizes decision-making power to lower levels and promotes proactive behaviors among employees to solve problems and take advantage of opportunities (Tatikonda and Rosenthal 2000). When employees perceive that they are empowered to make decisions or to conduct practices that benefit the company in which they work, they will tend to develop intrapreneurial and exploratory behaviours (Villiers-Sheepers 2012). Moreover, and because exploratory behaviors require non-routine problem solving and deviation from existing knowledge (Lavie et al. 2010), work discretion is likely to facilitate both incremental and discontinuous strategic corporate entrepreneurship practices.

Previous research suggests that employees with higher levels of autonomy tend to develop more creative ideas (Kuratko et al. 2014). In this regard, Hornsby et al. (2009) found that work discretion was positively and significantly correlated with the number of ideas implemented in the company, and Wyk and Adonisi (2012) found that work discretion was positively and significantly correlated with intrapreneurship. In accordance with the stated above, the second hypothesis is formulated as follows:

H2: Work discretion is positively related to incremental and discontinuous strategic corporate entrepreneurship practices.

### Rewards/reinforcements

The third factor of the internal organizational environment is rewards/reinforcements, which, when structured appropriately, can motivate employees to engage in innovative, proactive, and risk-taking behavior (Hornsby et al. 2009; Monsen et al. 2010). Since 1990, different authors have identified rewards as an organizational factor directly related to corporate entrepreneurship practices (Kuratko et al. 1990). Thanks to this type of pioneering work, it is now known that the appropriate use of rewards and reinforcements is a crucial organizational factor that allows the development of entrepreneurial and strategic behaviors in terms of innovation and proactivity (Monsen et al. 2010; Villiers-Sheepers 2012). An effective reward system, based on delineated goals, successive feedback, and different incentives such as those based on results, has been supported by an extensive literature that positively relates a comprehensive system of rewards with the development of strategic and entrepreneurial practices within companies (Hornsby et al. 2002). For instance, Hornsby et al. (2009) identified positive and significant correlations between rewards and the number of ideas implemented in the company. Wyk and Adonisi (2012) found that extrinsic job satisfaction, whose subscales included the reward systems, was positively and significantly correlated to intrapreneurship. For their part, Hornsby et al. (2013) also found significant correlations between rewards and corporate entrepreneurship. It is, therefore, hypothesized that:

H3: Rewards/reinforcements are positively related to incremental and discontinuous strategic corporate entrepreneurship practices.

## Methods

### Research design

The choice of conducting a survey was based on the appropriateness of this instrument to test causal relationships between a set of variables using a hypothetic-deductive methodology (Dana and Dana 2005). A literature review conducted previously (Casales Morici and Zander 2020) revealed that the analysis of different types and dimensions of strategic corporate entrepreneurship and the examination of their organizational antecedents is still limited. This was the primary motivation for the design of the study, which led to the testing of relationships through a series of hypotheses.

### Setting

The financial services sector offers an interesting arena for empirical work on corporate and strategic entrepreneurship, not only because it presents different conditions and outcomes from those presented by other industries (e.g., the manufacturing and high-technology industry), but also because it is a sector that has also changed dramatically during the past decades. The regulation of financial services has significantly increased in the last decade. Since the financial crisis in 2007–2008, an extensive supervisory and regulatory reform has been put forward in the European Union. Nevertheless, Europe’s financial systems' recent tendencies towards liberalization and deregulation have considerably influenced the increasing development of strategic and entrepreneurial practices within financial services firms. In this regard, established financial services firms that were once very traditional and conservative are now looking to adopt entrepreneurial practices that enable them to adapt to a fast-changing and tech-driven ecosystem to meet the rising expectations of a technologically inclined customer base. These challenges pressured on financial services firms to achieve renewal via strategic and entrepreneurial practices in the form of new products and services, organizational rejuvenation, and strategy renewal.

Nevertheless, the financial sector is well known for its difficulties with embedding emerging technologies to explore and exploit new business opportunities (Gomber et al. 2018; Tushman and O’Reilly 1996). It not only presents several internal barriers to entrepreneurship, such as restrictive mindsets, lack of qualified personnel, legacy systems, or unsupportive organizational structures (Das et al. 2017), but it also has various organizational designs depending on the nature of the tasks and practices that firms undertake (Falconer 2014). Moreover, in response to the global financial crisis of 2008, the sector was affected by new regulations that probably slowed the adoption of new technologies and the development of entrepreneurial practices within financial services firms.

Therefore, empirical research exploring different ways in which financial services firms could accelerate the development of innovations and entrepreneurial practices is needed. In this regard, examining how internal organizational conditions facilitate strategic corporate entrepreneurship can likely provide valuable and new insights.

### Sample

Data for the study were collected from employees at a large insurance company in Sweden. The insurance company offers services based on different combinations of non-life insurances, accident and medical insurances, life insurances, pension savings plans, and various banking services. At the time of the data collection, the insurance company consisted of 23 cooperating insurance companies, which had approximately 6,200 employees and 3.7 million customers. The final sample was drawn from two of these 23 cooperating insurance companies, each located in different regions of Sweden.

The first insurance company includes five offices placed in the north-eastern of Sweden (Sundsvall, Härnosand, Kramfors, Sollefteå, and Örnsköldsvik) and the second includes four offices ubicated in Stockholm (Frösunda, Globen, Stureplan, Norrtälje).

The company was selected for several reasons. First, during contact with the Centre for Research on Economic Relations at Mid Sweden University, company representatives expressed great interest in participating in the research. In particular, they described wanting to improve their organizational environment to facilitate the flow of new ideas within the company. Second, the company belongs to and operates in the financial sector, the amount of research on which, as explained earlier, pales in comparison to that in contexts other than manufacturing and technology-intensive industries. Furthermore, during the past few years, the insurance industry has been facing an increasingly dynamic environment. Rapid digitalization has forced incumbent firms to innovate and renew their traditional products and service offerings (Das et al. 2017). Third and last, the company is large. Although the study of entrepreneurship and renewal within companies is not limited to any particular type of organization, internal entrepreneurial processes described in existing literature are most likely found in larger companies.

### Data collection

The empirical investigation proceeded in two stages. The first stage involved four pilot interviews (that lasted about 45 min), conducted in November 2016, at one of the company offices to gain a better understanding of the company’s and employees’ perspectives on work environment at the office. Those semi structured interviews, together with the analyses conducted during the literature review, contributed to the development of items included in a subsequently administered questionnaire with reference to measures in the Corporate Entrepreneurship Assessment Instrument (CEAI). In the next phase, a survey was administered to employees in November 2017. Individualized links were e-mailed to each potential respondent, all of whom were asked to give answers on a 7-point Likert scale (1 = strongly disagree, 7 = strongly agree). To increase the response rate percentage, the questionnaire was sent in three waves, each about 2 weeks apart. The second wave was sent as a reminder to all respondents who had not submitted the questionnaire, followed by a final reminder to those who still had not responded. For confidentiality reasons, several offices included in the sample decided to exclude demographic information from the questionnaire (e.g., age, gender, and education). Consequently, it was not possible to include these variables in the statistical model. Ultimately, 194 responses were collected, of which 182 were valid^2^ and, therefore, included in the analysis.

### Method to estimate the sample size

Although determination of appropriate sample size is a critical issue in SEM, unfortunately, there is no consensus in the literature regarding what would be the appropriate sample size for SEM. Some evidence exists that simple SEM models could be meaningfully tested even if sample size is quite small (Hoyle 1999; Hoyle and Kenny 1999), but usually, *N* = 100–150 is considered the minimum sample size for conducting SEM (Tinsley and Tinsley 1986; Anderson and Gerbing 1988; Ding et al. 1995; Tabachnick and Fidell 2013). Simulation studies show that with normally distributed indicator variables and no missing data, a reasonable sample size for a simple CFA model is about *N* = 150 (Muthén and Muthén 2002). I have relied on two “rules of thumb”. The first is the observation-to-variable ratio of 20:1 (Hair et al. 2019). This means that though a minimum of 20 respondents must be considered for each independent variable in the model. In addition, I also followed the formula of Tabachnick and Fidel (2013, p.123), *N* > 50 + 8*m* (where *m* is the number of independent variables).

### Measures

#### Scale development theoretical/methodological decision measures

After an extensive literature review (see Casales Morici and Zander 2020) no instruments for identifying strategic corporate entrepreneurship practices were found (i.e., instruments that separately capture the various forms through which strategic entrepreneurship manifests). The extant literature in strategic corporate entrepreneurship is still at the beginning of applying differentiated measures and considering how these may relate to different aspects of the internal organizational environment. Some studies have used proxies (Brown et al. 2001; Kyrgidou and Petridou 2011) but they are insufficient, as they do not capture the different forms that strategic corporate entrepreneurship practices can take within companies. Only one study attempting to develop a measure of strategic entrepreneurship construct was found (Kantur 2016). Therefore, several items were created to develop the constructs to measure strategic corporate entrepreneurship practices. Scale items creation of the dependent variables, i.e., strategic corporate entrepreneurship practices in their incremental and discontinuous forms, relied on the above-mentioned literature review. The literature review on strategic entrepreneurship played the strongest role in guiding the identification of empirical attributes that represent the constructs (Clark and Watson 2016; DeVellis 2012). Six items were generated to capture the three forms of strategic entrepreneurship: the introduction of new products or services, organizational rejuvenation, and strategic renewal; in their different nature, i.e., incremental, and discontinuous. A complete version of the questionnaire was presented in a seminar at Uppsala University, in which a group of researchers and experts in corporate entrepreneurship assessed the degree to which the items reflect the theoretical content domain. They assessed item validity through open-ended feedback (DeVellis 2012; Ruel et al. 2016) and suggested that items sample the domains they are intended to capture without measuring other domain(s).

The following three items formed the dependent variable reflecting discontinuous strategic corporate entrepreneurship practices:

1. *Sustained regeneration* The products and services our company develops are often pioneering and new to the world.
2. *Organizational rejuvenation* Our company often faces radical changes in the way we work.^3^
3. *Strategic renewal* In our company, we often introduce radical changes to the existing strategy.^4^

Three items equally formed the dependent variable reflecting the incremental strategic corporate entrepreneurship:

1. *Sustained regeneration* Our company often develops products or services based on our existing product and service portfolio.
2. *Organizational rejuvenation* In our company, we often make small changes in the way we work.
3. *Strategic renewal* In our company, we often make minor adjustments to the existing strategy.

### Validity and reliability of constructs

#### Dependent variables

To ascertain whether there were two separate factors and that the items loaded appropriately, the six items were subjected to principal component analysis. Kaiser’s criterion and parallel analysis were used to assist in the decision concerning the number of factors to retain. Prior to performing it, the suitability of data for factor analysis was assessed. Inspection of the correlation matrix revealed the presence of coefficients of 0.3 or above. The Kaiser–Meyer–Olkin value was 0.702, exceeding the recommended value of 0.6 (Kaiser 1970, 1974) and Bartlett’s test of sphericity (Bartlett 1954) reached statistical significance, supporting the factorability of the correlation matrix. The principal component analysis method revealed a two-component solution that explained a total of 66% of the variance, with component 1 (discontinuous strategic corporate entrepreneurship) contributing 42% and component 2 (incremental strategic corporate entrepreneurship) 23%. To aid in the interpretation of these two components, Oblimin rotation was performed. The rotated solution revealed a simple structure (Thurstone 1947), with both components showing a number of solid loadings above 0.7. The reliability was appraised using three indicators: Cronbach's *α*, AVE, and the square root of AVE (composite reliability), as shown in Table 1.

**Table 1:** Cronbach's *α*, AVE, and the square root of AVE (composite reliability) of each construct

Constructs	Cronbach's *α*	AVE	The square root of AVE
1. Management support (MS)	0.733	0.53	0.77
2. Work discretion (WD)	0.808	0.63	0.84
3. Rewards/reinforcement (RR)	0.674	0.55	0.70
4. Discontinuous SCE	0.753	0.59	0.81
5. Incremental SCE	0.742	0.53	0.77

#### Independent variables

The CEAI’s content, as well as construct and convergent validity, have been assessed by Hornsby et al. (2013), whose factor analysis supported the existence of four of five originally proposed factors (i.e., management support, work discretion, reward/reinforcement, and time availability). From the original 48 items, only 18 remained after their analysis. Following the recommendation of Hornsby et al. (2013), those 18 items were included in the questionnaire. To ascertain whether there were three separate factors and that the items loaded appropriately, exploratory factor analysis was conducted, and the items were subjected to principal component analysis. Kaiser’s criterion and Parallel Analysis were used to assist in the decision concerning the number of factors to retain. Because the fourth factor's reliability (time availability) did not meet the 0.50 threshold (Nunnally 1978), this variable was dropped from the analysis.^5^ To aid in the interpretation of the components, Oblimin rotation was performed. The rotated solution revealed the presence of a simple structure (Thurstone 1947), considering only components loading above 0.7 (Hair et al. 2014). Appendix 1 shows the components and their loadings.

Cronbach’s α coefficients were calculated to assess the internal consistency of the variables. Ranging from 0.67 to 0.81, the coefficients exceeded the minimum threshold and could be considered acceptable to good (Nunnally 1978). The AVEs of the relationship between management support, work discretion, rewards/reinforcements, discontinuous, and incremental strategic corporate entrepreneurship were 0.53, 0.63, 0.55, 0.59, and 0.53, respectively. The composite reliability was assessed by analyzing the standardized loadings, which should be equal to or greater than 0.7. The composite reliability for the constructs were: management support 0.77; work discretion 0.84; rewards/reinforcements 0.7; discontinuous strategic corporate entrepreneurship 0.81, and incremental strategic corporate entrepreneurship 0.77.

### Assessing common method variance

Two approaches for addressing common method effects have been conducted: preventive (procedurally) and detective (statistically). As a preventive technique I have applied the procedural remedies recommended by Podsakoff et al. (2003). Both the pilot study and the questionnaire implemented procedural remedies to reduce common bias, such as ensuring the anonymity of respondents, providing contextual information and definitions to reduce ambiguities, and assuring respondents that there were no right or wrong answers (Podsakoff et al. 2003). As a detective technique and because the questionnaire was based on self-reported information, common method variance was investigated using Harman’s Single Factor test to address this issue. Applying the approach described in Podsakoff et al. (2003), the raw data (including the dependent variables) were analyzed through exploratory factor analysis and inspection of the unrotated factor solution. The factor analysis generated four factors, with the first and second factors accounting for less than half of the covariance among the measures. The absence of a single factor emerging, or one factor accounting for a majority of the covariance, suggests that common method bias is unlikely to have had a decisive effect on the results. Table 2 presents the descriptive statistics and the correlation matrix.

**Table 2:** Means, standard deviations, and correlation matrix *p < 0.05 **p < 0.01

Constructs	Means	Standard deviation	1	2	3	4
1. Management support	4.79	1.217**	1.000			
2. Work discretion	5.03	1.390**	0.295**	1.000		
3. Rewards/reinforcement	4.52	0.921**	0.131	0.289**	1.000	
4. Discontinuous SCE	4.68	1.234**	0.323**	0.396**	0.360**	1.000
5. Incremental SCE	4.09	1.150**	0.409**	0.026	0.168*	0.262**

## Data analysis

### The structural equation modelling

**Fig. 1:**
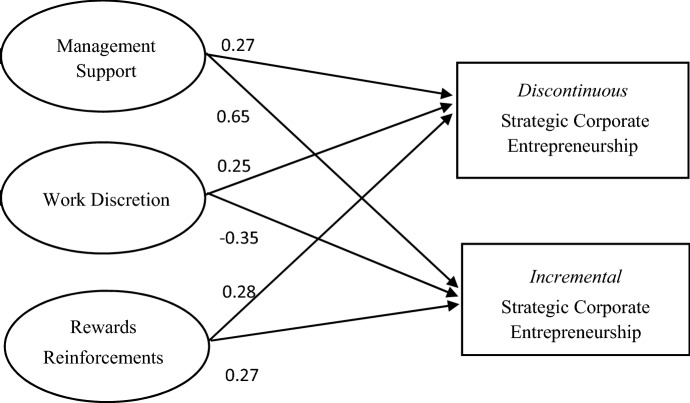
The SEM for discontinuous and incremental strategic corporate entrepreneurship and the organizational antecedents

To assess the validity and reliability of the measurement model, the procedures recommended by Anderson and Gerbing (1988) were followed. Appendix 2 provides a detailed explanation of the procedures followed to assess the discriminant validity. To test for associations between the internal organizational factors and strategic corporate entrepreneurship practices, the hypothesized Structural Equation Model was performed using LISREL 8.80 (Jöreskog and Sörbom 2007). The maximum likelihood estimation method was applied to test the model, using the correlation matrix and covariance matrix as input. This type of analysis can correct the unreliability of measures and gives information on the paths between the multiple constructs. Figure 1 shows that the exogenous constructs of organizational factors influence the endogenous variables of discontinuous and incremental strategic corporate entrepreneurship.

The hypotheses H1 and H3 were supported. Multiple fit indices were used to evaluate fit so that judgments are not an artifact of analytical choice. The overall model showed values for GFI, NFI, NNFI, and CFI of 0.91, 0.95, 0.98, and 0.99, respectively. These values exceed the recommended threshold of 0.90. Finally, the RMSEA value of the overall model was 0.038, below the recommended threshold value of 0.10 (Hair et al. 2014). To summarize, all fit indices indicated exceed the recommended guidelines for good fit; therefore, the model reflects good measurement and statistical fit. The results of the structural model are presented in Table 3.

**Table 3:** Results from the structural model

Relations	Effect	Path coefficient (standardized solution)	*t* value
H1a: management support—discontinuous SCE	+	0.27	2.81****
H1b: management support—incremental SCE	+	0.65	4.28****
H2a: work discretion—discontinuous SCE	+	0.25	2.26*
H2b: work discretion—incremental SCE	**–**	− 0.35	− 2.53***
H3a: rewards/reinforcement—discontinuous SCE	+	0.28	2.27*
H3b: rewards/reinforcement—incremental SCE	+	0.27	1.98*

*t values > 1.96 = level of significance 0.05

## Discussion

This study furnishes novel empirical evidence of organizational antecedents supporting strategic corporate entrepreneurship practices in the often-overlooked financial service sector. Breaking down strategic corporate entrepreneurship into its different practices and natures, the study offers a foundation for developing a more generally applicable measure of the concept. By exploring potentially differentiated effects of internal organizational factors such as management support, work discretion, and rewards/reinforcement, this study demonstrated how incremental and discontinuous corporate strategic entrepreneurship practices are influenced by  the company’s internal organizational environment. While the factors *management support* and *rewards/reinforcement* present the same effect, work discretion shows differentiated effects on discontinuous versus incremental strategic corporate entrepreneurship practices. The findings support the argument about the need for and importance of creating an “organizational climate” that facilitates the development of both discontinuous and incremental practices (Ireland and Webb 2007). Although some internal organizational factors are drivers of discontinuous strategic corporate entrepreneurship practices, others are facilitators of incremental ones. This was especially reflected in the organizational factor called work discretion (i.e., decentralized authority). When developing and conducting strategic corporate entrepreneurship practices of a different nature (i.e., discontinuous and incremental), firms have the difficult task of creating an organizational design aimed to facilitate two opposite but complementary objectives. Employees must have a certain level of autonomy to make decisions about performing their work in the way that they believe is most effective. This leads to proactive behaviors to solve problems, pursue opportunities, and encourage creativity and exploration (Ireland and Webb 2007). However, some degree of standardization and fixed processes are required to reduce employees’ autonomy and enhance stability to prioritize future revenues, maintain their current competitive advantages and allow regulatory readiness.

In addition, the findings revealed a correlation between *management support* for incremental practices stronger than for discontinuous practices. This can be explained by the fact that incremental practices are more common in established financial services firms. For decades these firms could rely on mere incremental improvements of their service offerings (Berry et al. 2006). In this respect, managers and their supporting behaviors toward incremental activities could empower the whole organization to cope with changes as they occur, facilitating the accumulation of small adjustments leading to major transformations in the long run. This also provides the ability to incorporate new routines and processes for facilitating change in the firm’s structural inertia (Amburgey et al. 1993).

The results further highlight the use of different sources of *rewards and reinforcements* as a driver of both discontinuous and incremental practices. Overall, these managerial dynamics represent an important piece of information for financial services firms characterized by inflexible and inappropriate management systems, where a focus on policy and compliance dominates the firm’s managerial thinking (Naylor 2017). High levels of organizational flexibility coupled with a culture prepared to address change are perceived to be even more critical when the forces driving the change are intensified (Grewal and Tansuhaj 2001).

## Implications

### Theoretical

This thesis offers new theoretical insights through the lens of strategic corporate entrepreneurship conducted by firms operating in highly regulated sectors manufacturing not commonly found in strategic entrepreneurship and innovation literature. These insights contribute to the development of more contextually sensitive theories and conceptual models (cf. Hughes and Mustafa 2017; Kyrgidou and Petridou 2011; Kantur 2016), highlighting the boundary conditions of theories and models across different industries and institutional settings. It is, thus, in line with recent suggestions about developing a greater sensitivity to how strategic corporate entrepreneurship unfolds, depending on the different contexts and sectors in which the firm operates (Bruton et al. 2008; Hitt et al. 2011; Sakhdari et al. 2014; Welter 2011; Zahra 2007). Therefore, this study offers an important first step toward understanding the organizational factors that spur employees to undertake different strategic corporate entrepreneurship practices. To the best of the author’s knowledge, it is the first study that has empirically tested the relationships between factors such as management support, work discretion, and rewards/reinforcements and strategic corporate entrepreneurship practices in their incremental and discontinuous forms.

### Methodological

From a methodological view, this study clarified and differentiated strategic corporate entrepreneurship practices by breaking them into three types (i.e., sustained regeneration, organizational rejuvenation, and strategic renewal) and their natures (i.e., discontinuous, and incremental). Until now, strategic corporate entrepreneurship has been mainly envisioned and empirically tested as a homogenous phenomenon, ignoring the variety of activities and practices it includes and the nature of those. By developing a potential measure of strategic corporate entrepreneurship practices, this study offers an important tool that can help the strategic entrepreneurship field advance stronger and more reliable for its theoretical claims.

### Managerial

This study ultimately enhances and enriches the strategic and entrepreneurial toolbox for practicing managers and policymakers. First, it provides a deeper understanding of how an appropriate design of internal organizational resources can prepare the ground for strategic corporate entrepreneurship practices, offering managers a foundation on which to base their decisions. In this regard, the findings suggest that organizational factors such as management support, work discretion, and rewards/reinforcement play an essential role in empowering employees to conduct both incremental and discontinuous practices. Thus, the study guides corporate managers and leaders of financial services firms interested in motivating their employees to undertake those practices. Specifically, the findings represent an important piece of information for financial services firms, which are often characterized as being inflexible and using inappropriate management systems (Naylor 2017). Considering this, the findings highlight that the biggest hurdles to the effective development of entrepreneurial and strategic practices within financial services firms can be given by the lack of support from managers to champion new ideas suggested by employees in a bottom-up manner. Moreover, the differentiated effects of work discretion in incremental and discontinuous practices also represent a critical managerial implication. On the one hand, centralization is needed for incremental strategic corporate entrepreneurship practices as it defines a clear locus of control and minimizes deviations from rules and procedures by communicating clearly “what to do” (Cardinal 2001). In turn, decentralization is also needed for discontinuous strategic corporate entrepreneurship practices as it removes restrictions on employees by encouraging employee autonomy, creativity, exploration, and entrepreneurial behaviors, which ultimately provides firms with new opportunities (Ireland and Webb 2007). In line with this, a recent study showed that only 5% of the middle managers in insurance companies felt that senior managers understood the organizational changes required to conduct entrepreneurial practices (Ernst and Young 2013). While 30% envisaged improvements, no insurance company had senior managers that seemed to comprehend the scale and urgency of the transformation needed. Considering this, the biggest hurdles to the effective development of strategic corporate entrepreneurship practices within financial services firms may not be the lack of strategy or availability of resources but rather the need for more support and engagement from managers. In this regard, the study shows the importance of having supportive managers that motivate and incentivize employees to conduct strategic corporate entrepreneurship practices. Without this important organizational driver, established financial services firms would continue to lag further behind other sectors, remaining thus highly vulnerable to the entry of external disruptors.

## Limitations and future research

Caution must be exercised, of course, when generalizing the results. The study has focused on internal organizational factors and how they may be associated with strategic corporate entrepreneurship practices. Therefore, it has been a limited reflection on how the context could influence the design and relationships between the organizational factors examined in this study. However, it has been shown that new legislation resulting from the global financial crisis of 2008 and the tendencies of Europe’s financial systems towards liberalization and re-regulation required firms operating in the financial sector to reassess their organizational structures, strategies, processes, and operations (Das et al. 2017). Such firms were forced to leverage “new to the firm” capabilities, create adapted organizational structures, and embed processes to enable innovation. Consequently, large and established financial services firms cautiously forecasted their role in implementing different courses of action to enhance their organizational innovative capacity (Das et al. 2017). In the years after this study was conducted, different phenomena (digitalization, Covid pandemic, etc.) have affected the financial and other sectors in different ways. Therefore, it would be beneficial if future research could compare, for instance, the organizational impact before and after the COVID pandemic. It could also be investigated if the incentives would be the same or if there would be less interest in incentives and more in redefining the core business for companies hardly hit by COVID-19.

Another limitation was the requirement placed by the company about restrictions on data collection. Although it would have been preferred to have a separate measurement for the dependent and independent variables at different points of time, the concerns relating to the time burden on employees called for a survey conducted in one wave. In addition, company restrictions prevented the inclusion of an expanded number of items as well as demographic information.

This study leaves a number of questions open for further investigation. First, the limited amount of empirical research on strategic corporate entrepreneurship remains a concern, especially in the financial sector. While this study has initiated the clarification and refinement of the relationship between internal organizational factors and discontinuous and incremental strategic corporate entrepreneurship practices, further research could usefully consider and test relationships between these organizational factors and other strategic corporate entrepreneurship practices. Furthermore, studies with a qualitative nature, such as case studies, would be a fruitful further step toward a better understanding of the dynamics involved in these practices.

Furthermore, there were no (and still aren’t) accepted measures of strategic corporate entrepreneurship. Thus, a validated instrument that separately captures the various practices of strategic corporate entrepreneurship in its different forms is needed. By developing more rigorous approaches to measuring strategic corporate entrepreneurship practices, the field will advance stronger and with more reliable support for its theoretical claims. Finally, industry structure, sector characteristics and national contexts might have an important impact on the volume and nature of opportunities surrounding companies and could motivate or discourage strategic corporate entrepreneurship practices accordingly. Further explorations of the impact of industry and country-specific organizational features could unearth some important industry-dependent and international differences in the sources, processes, and outcomes of strategic corporate entrepreneurship practices (Zahra 1991).

## Conclusions

This study has empirically shown that financial services firms can use their internal organizational resources to facilitate the development of  strategic corporate entrepreneurship practices of different nature (i.e. incremental and discontinuous), by adapting the design of those organizational resources. The study contributes to both strategic corporate entrepreneurship and financial services firms’ literature in at least three ways. First, it illustrates the need for an adapted organizational structure with internal organizational resources that facilitate both types of activities (Ireland and Webb 2007). This study, therefore, takes a preliminary step toward addressing the association of organizational factors such as management support, work discretion, and rewards/reinforcements with different strategic corporate entrepreneurship practices such as sustained regeneration, organizational rejuvenation, and strategic renewal. To the best of the author’s knowledge, it is the first study that has empirically tested these relationships in financial services firms. This allows scholars to develop a greater sensitivity to how strategic corporate entrepreneurship unfolds, depending on the different contexts and sectors in which the firm operates. Second, by exploring how the internal organizational environment can be designed to facilitate strategic corporate entrepreneurship practices, the study also offers managers a solid foundation to base their decisions. This was especially reflected in the organizational factor work discretion. Whereas discontinuous strategic corporate entrepreneurship practices require a decentralized organizational design, where creativity and innovation are supported and championed, incremental practices need more rigid organizational designs facilitating control, stability, and predictability through a centralized authority. Third, as a methodological contribution, the study pioneers the clarification and differentiation of strategic corporate entrepreneurship practices, breaking them down into their different practices and natures. This represent an important contribution given that yet, strategic corporate entrepreneurship has been mainly envisioned and empirically tested as a homogenous phenomenon. The results suggest that viewing strategic corporate entrepreneurship as a set of practices that are organizationally homogeneous is unduly restrictive. Instead, embracing it as a heterogeneous phenomenon can provide additional richness and depth, not only to the strategic corporate entrepreneurship literature but also to develop complementary process models in the broader literature of financial service firms. 

Despite the importance of the financial service sector in most economies, how strategic corporate entrepreneurship unfolds in these firms is still not well understood. The design of entrepreneurial organizational factors provides tantalizing possibilities of how strategic corporate entrepreneurship is manifested. Overall, the findings not only highlight the efforts of financial services firms to increase the development of strategic corporate entrepreneurship practices but also the need to devise internal organizational mechanisms such as the support from managers and employee autonomy to stimulate those practices within the firm.

## Supplementary Information

Below is the link to the electronic supplementary material.

Supplementary file1 (DOCX 54 KB)

## Data Availability

The data sets generated during and/or analyzed during the current study are available in the Open Science Framework (OSF) repository: https://mfr.osf.io/render?url=https://osf.io/4jahz/?direct%26mode=render%26action=download%26mode=render.

## Footnotes

1 There are many different uses of the term “strategic renewal” in the literature. In this study, and in line with Covin and Miles (1999), the term refers to the implementation of a “new” business strategy.

2 Twelve responses were excluded because they presented missing data.

3 Incremental organizational rejuvenation was defined as a series of regular minor adjustments in the way in which employees work, involving a gradual and adaptive process, adding, if necessary, complementary tasks that maintain the main firm’s operations. On the other hand, discontinuous organizational rejuvenation refers to reconfigurations of intern processes, main operations of the firm, and administrative systems.

4 The company´s strategy was defined here as the refreshment (incremental) or replacement (discontinuous) of the key ideas and/or main vision of the company (Guth & Ginsberg, 1990).

5 The instability of the time availability construct was somewhat consistent with previous efforts, where the CEAI had been applied. For instance, in the study where the CEAI was re-assessed (Hornsby, Kuratko, Holt & Wales, 2013), this construct revealed a weak relationship to entrepreneurial orientation. It showed neither content validity nor reliability when it was examined in the context of emerging markets (Bhardwaj & Sushil, 2012; De Villiers–Scheepers, 2012). Similarly, Hornsby et al. (2009) detected a negative correlation between employees’ time availability and the number of implemented ideas. Furthermore, in the study conducted by Van Wyk and Adonisi (2012), where the relationships between the different constructs of market orientation, flexibility, and job satisfaction with the CEAI constructs were examined, the construct time availability was the only factor that did not present a significant correlation or prediction.

## Declarations

### Author information

Affiliations: Department of Business Studies, Uppsala University, Sweden. Procome research group, Department of Learning, Informatics, Management and Ethics, Medical Management Centre, Karolinska Institutet, Stockholm, Sweden.
