# Supplementary material for: Strategic corporate entrepreneurship practices in financial services firms: the role of organizational factors
Source: SN Bus Econ. 2022 Aug 13;2(9):130. doi: 10.1007/s43546-022-00306-2 (PMC9376042; doi:10.1007/s43546-022-00306-2)
Supplement: Supplementary file 1 — Supplementary file1 (DOCX 54 KB) [file 43546_2022_306_MOESM1_ESM.docx]

**Appendix 1: Factor analysis**

**Independent variables**

| **Item** | **Pattern coefficients** | | | **Structure coefficients** | | | **Communalities** |
| --- | --- | --- | --- | --- | --- | --- | --- |
|  | **Management Support** | **Work Discretion** | **Rewards**  **Reinforcement** | **Management Support** | **Work Discretion** | **Rewards**  **Reinforcement** |  |
| **Q1** | **.773** | .030 | -.063 | **.774** | .174 | -.010 | **.604** |
| **Q2** | **.786** | .065 | -.103 | **.791** | .211 | -.050 | **.640** |
| **Q3** | **.747** | .202 | -.128 | **.776** | .340 | -.076 | **.658** |
| **Q4** | -.114 | -.079 | .219 | -.113 | -.099 | .211 | .067 |
| **Q5** | .082 | -.012 | -.170 | .068 | .003 | -.165 | .034 |
| **Q6** | .121 | **.820** | .052 | .277 | **.843** | .063 | **.728** |
| **Q7** | .060 | **.833** | .119 | .223 | **.844** | .127 | **.731** |
| **Q8** | .019 | **.759** | .207 | .175 | **.764** | .212 | **.627** |
| **Q9** | -.167 | .385 | -.276 | -.114 | .353 | -.286 | .234 |
| **Q10** | .283 | -.093 | .078 | .271 | -.039 | .097 | .088 |
| **Q11** | .116 | .285 | **.766** | .221 | .310 | **.776** | **.708** |
| **Q12** | .140 | .055 | **.790** | .204 | .084 | **.800** | **.665** |
| **Q13** | .217 | .110 | .278 | .257 | .151 | .293 | .154 |

**Dependent variables**

|  | **Pattern coefficients** | | **Structure coefficients** | | **Communalities** |
| --- | --- | --- | --- | --- | --- |
|  | **Discontinuous SCE** | **Incremental SCE** | **Discontinuous SCE** | **Incremental SCE** |  |
| **Q14** | **.883** | -.074 | **.862** | .175 | .748 |
| **Q15** | **.862** | -.026 | **.855** | .217 | .731 |
| **Q16** | **.700** | .116 | **.733** | .314 | .550 |
| **Q17** | -.028 | **.767** | .189 | **.759** | .576 |
| **Q18** | .013 | **.854** | .254 | **.857** | .735 |
| **Q19** | .025 | **.810** | .253 | **.817** | .667 |

**Appendix 2: Discriminant validity - procedures** **(Anderson and Gerbing, 1988)**

1) *Convergent and discriminant validity:*

The *discriminant validity* was assessed through the analysis of the Phi covariance matrix and the Phi correlation matrix.

Phi Covariance Matrix (std. errors): covariances between the latent variables

|  | Management Support | Work Discretion | Rewards/Reinforcement |
| --- | --- | --- | --- |
| Management Support | 1 |  |  |
| Work Discretion | 0.24 (0.06) | 1 |  |
| Rewards/Reinforcement | 0.15 (0.06) | 0.33 (0.06) | 1 |

Phi Correlation Matrix: correlations between the latent variables

|  | Management Support | Work Discretion | Rewards/Reinforcement |
| --- | --- | --- | --- |
| Management Support | 1 |  |  |
| Work Discretion | 0.43 | 1 |  |
| Rewards/Reinforcement | 0.26 | 0.47 | 1 |

Anderson and Gerbing (1988): in the Phi Correlation Matrix, the loading correlations +/- (2 x std. error) = the resulting number does not include 1.

**Management Support to Work Discretion**

2 x 0.06 = 0.12

0.31-----0.43-----0.55

This is a confidence interval around 0.43

(It does not go below -1 or above 1, so it does not include 1. It presents discriminant validity)

0.43 + 0.12 = 0.55

0.43 – 0.12 = 0.31

**Management Support to Rewards/Reinforcement**

2 x 0.06 = 0.12

0.14-----0.26-----0.38

This is a confidence interval around 0.26

0.26 + 0.12 = 0.38

0.26 – 0.12 = 0.14

**Work Discretion to Rewards/Reinforcement**

2 x 0.06 = 0.12

0.35-----0.47-----0.59

This is a confidence interval around 0.47

0.47 + 0.12 = 0.59

0.47 – 0.12 = 0.35

**Appendix 3:** **Variance extracted and composite reliability - procedures (Fornell and Larcker, 1981)**

| LAMBDA-X (standardized loadings) | | | | | |
| --- | --- | --- | --- | --- | --- |
|  | Management Support | Work Discretion | Rewards / Reinforcement | Discontinuous | Incremental |
| Q1 | 0.67 |  |  |  |  |
| Q2 | 0.78 |  |  |  |  |
| Q3 | 0.72 |  |  |  |  |
| Q4 |  | 0.83 |  |  |  |
| Q5 |  | 0.88 |  |  |  |
| Q6 |  | 0.67 |  |  |  |
| Q7 |  |  | 0.84 |  |  |
| Q8 |  |  | 0.67 |  |  |
| Q9 |  |  |  | 0.83 |  |
| Q10 |  |  |  | 0.82 |  |
| Q11 |  |  |  | 0.64 |  |
| Q12 |  |  |  |  | 0.65 |
| Q13 |  |  |  |  | 0.82 |
| Q14 |  |  |  |  | 0.70 |
| THETA DELTA | | | | | |
|  | Management Support | Work Discretion | Rewards / Reinforcement | Discontinuous | Incremental |
| Q1 | 0.55 |  |  |  |  |
| Q2 | 0.38 |  |  |  |  |
| Q3 | 0.48 |  |  |  |  |
| Q4 |  | 0.32 |  |  |  |
| Q5 |  | 0.23 |  |  |  |
| Q6 |  | 0.56 |  |  |  |
| Q7 |  |  | 0.39 |  |  |
| Q8 |  |  | 0.57 |  |  |
| Q9 |  |  |  | 0.32 |  |
| Q10 |  |  |  | 0.32 |  |
| Q11 |  |  |  | 0.59 |  |
| Q12 |  |  |  |  | 0.58 |
| Q13 |  |  |  |  | 0.32 |
| Q14 |  |  |  |  | 0.51 |
| PARAMETER SUM | | | | | |
|  | Management Support | Work Discretion | Rewards / Reinforcement | Discontinuous | Incremental |
|  | 2.17 | 2.38 | 1.51 | 2.29 | 2.17 |
| PARAMETER SUM SQR | | | | | |
|  | Management Support | Work Discretion | Rewards / Reinforcement | Discontinuous | Incremental |
|  | 1.58 | 1.91 | 1.15 | 1.77 | 1.58 |
| THETA DELTA SUM | | | | | |
|  | Management Support | Work Discretion | Rewards / Reinforcement | Discontinuous | Incremental |
|  | 1.41 | 1.11 | 0.96 | 1.23 | 1.41 |
| COMPOSITE RELIABILITY (Cutoff >.7) | | | | | |
|  | Management Support | Work Discretion | Rewards / Reinforcement | Discontinuous | Incremental |
|  | 0.77 | 0.84 | 0.70 | 0.81 | 0.77 |
| VARIANCE EXTRACTED (Cutoff >.5) | | | | | |
|  | Management Support | Work Discretion | Rewards / Reinforcement | Discontinuous | Incremental |
|  | 0.53 | 0.63 | 0.55 | 0.59 | 0.53 |
